# Supplementary material for: Possible Causal Association between Type 2 Diabetes and Glycaemic Traits in Primary Open-Angle Glaucoma: A Two-Sample Mendelian Randomisation Study
Source: Biomedicines. 2024 Apr 15;12(4):866. doi: 10.3390/biomedicines12040866 (PMC11048047; doi:10.3390/biomedicines12040866)
Supplement: Supplementary file 1 [file biomedicines-12-00866-s001.zip › Additional file S1 final.pdf]

Possible Causal Association between Type 2 Diabetes and Glycaemic Traits in Primary Open-Angle Glaucoma: A Two-Sample Mendelian Randomisation Study

To reduce of concern according to population stratification, and enhance more robustly acknowledge, we performed additional analysis for association of type 2 diabetes and glaucoma as Table S2. As for the data set, Finngen and UKB composed of European results were appropriately used. Type 2 diabetes are coding as 'type 2 diabetes with other specified/multiple/unspecified complications' and 'type 2 diabetes, definitions combined' in Finngen. The authors will use the term 'type 2 diabetes with complications' for 'type 2 diabetes with other specified/multiple/unspecified complications' and 'type 2 diabetes ' to refer to 'type 2 diabetes, definitions combined' in this paper.

Table S2. Summary statistics of data source

**Traits**	**Data source**	**No. of participants**	**Population**	**No. of Variants**	**Reference**
Type 2 diabetes with complication	Finngen	354,653 (46,373 cases + 308,280 controls)	European	20,169,746	https://finngen.gitbook.io/documentation/v/r5/
Type 2 diabetes	Finngen	365,950 (57,698 cases + 308,252 controls)	European	20,170,006	https://finngen.gitbook.io/documentation/v/r5/
Glaucoma	UK biobank	456,348 (654 cases + 455,694 controls)	European	11,831,932	[66]

In addition, heterogeneity and horizontal pleiotropy of instrumental variables were described as Table S3. When interpreting these results, it was found that the MR results (exposure: type 2 diabetes with complication, or type 2 diabetes; outcome: glaucoma) should be interpreted focusing on the MR IVW method is ideal.

Table S3. Heterogeneity and horizontal pleiotropy of instrumental variables

**Exposure**	**Outcome**				**Heterogeneity**		**Horizontal pleiotropy**				
								**MR-Egger**		**MR-Egger (SIMEX)**	
		**N**	**F**	**I^2 (%)**	***p****	***p***†	***p***‡	**Intercept, β (SE)**	***p***	**Intercept, β (SE)**	***p***
Type 2 diabetes with complication	Glaucoma	157	65.01	94.60	0.417	0.425	0.407	-0.015 (0.013)	0.242	-0.017 (0.013)	0.198
Type 2 diabetes	Glaucoma	155	67.83	94.74	0.161	0.175	0.151	-0.018 (0.013)	0.169	-0.02 (0.014)	0.148

N, number of instruments; F, mean F statistic; IVW, inverse-variance weight; MR, mendelian randomization; PRESSO, pleiotropy residual sum and outlier; SIMEX, simulation extrapolation; β, beta coefficient; SE, standard error *Cochran's Q test from inverse-variance weight, † Rucker's Q' test from MR-Egger, ‡MR-pleiotropy residual sum and outlier global test

The results of MR analysis performed in the similar method as in the main text were obtained (**Fig S1**). Significant causal associations of 'type 2 diabetes with complication' on 'glaucoma' were observed (Odd ratio (OR): 1.24, *P*=0.006 in IVW, OR: 1.33, *P*=0.033 in MR weighted median, OR: 1.49, *P*=0.025 in MR Egger, and OR: 1.55, *P*=0.023 in MR Egger (SIMEX), **Fig S1 and Table S4**). In addition, significant causal associations of 'type 2 diabetes' on 'glaucoma' were observed (OR: 1.19, *P*=0.045 in IVW, OR: 1.36, *P*=0.029 in MR weighted median, OR: 1.52, *P*=0.035 in MR Egger, and OR: 1.57, *P*=0.034 in MR Egger (SIMEX), **Figure S1 and Table S4**).

Figure S1. Forest plot for association of type 2 diabetes and glaucoma

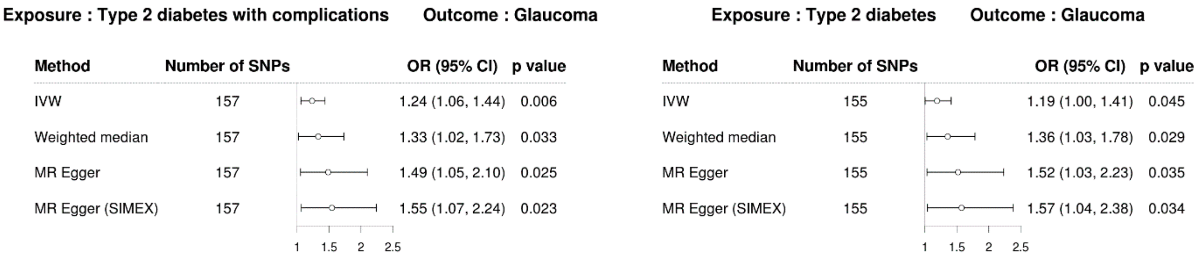

Table S4. Estimates from MR methods for the association between type 2 diabetes and glaucoma

Exposure	Outcome	Methods	Parameter	N	Odds ratio (95% CI)	p-value
Type 2 diabetes with complication	Glaucoma					
		IVW	Estimate	157	1.24 (1.06, 1.44)	0.006
		Weighted median	Estimate		1.33 (1.02, 1.73)	0.033
		MR-Egger	Intercept		0.99 (0.96, 1.01)	0.242
			Slope		1.49 (1.05, 2.10)	0.025
		MR-Egger (SIMEX)	Intercept		0.98 (0.96, 1.01)	0.198
			Slope		1.55 (1.07, 2.24)	0.023
		MR-PRESSO				
Type 2 diabetes	Glaucoma		Estimate	155	1.19 (1.00, 1.41)	0.045
		IVW	Estimate		1.36 (1.03, 1.78)	0.029
		Weighted median	Intercept		0.98 (0.96, 1.01)	0.169
		MR-Egger	Slope		1.52 (1.03, 2.23)	0.035
			Intercept		0.98 (0.95, 1.01)	0.148
		MR-Egger (SIMEX)	Slope		1.57 (1.04, 2.38)	0.034
			Estimate	157	1.24 (1.06, 1.44)	0.006
		MR-PRESSO	Estimate		1.33 (1.02, 1.73)	0.033

The genetic connection between type 2 diabetes effects on glaucoma were significant positive correlation (**Figure S2**).

Figure S2. Scatter plots of MR tests assessing the type 2 diabetes and glaucoma

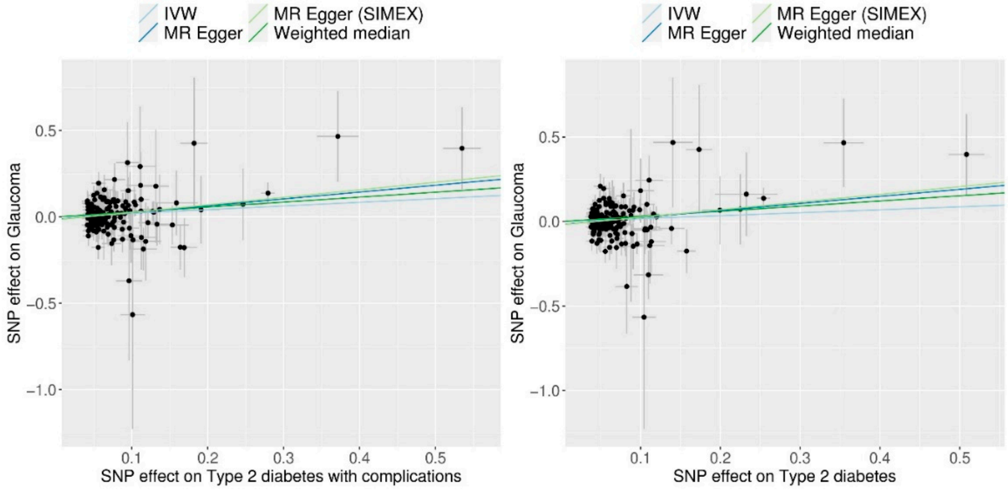

Light blue, light green, dark blue, and dark green regression lines represent the IVW, MR–Egger (SIMEX), MR–Egger, and weighted median estimate, respectively. MR, Mendelian randomization; SNP, single nucleotide polymorphism; IVW, inverse-variance weight; SIMEX, simulation extrapolation
